# Inferring gene and protein interactions using PubMed citations and consensus Bayesian networks

**DOI:** 10.1371/journal.pone.0186004

**Published:** 2017-10-19

**Authors:** Anthony Deeter, Mark Dalman, Joseph Haddad, Zhong-Hui Duan

**Affiliations:** 1 Integrated Bioscience, University of Akron, Akron, Ohio, United States of America; 2 Department of Computer Science, University of Akron, Akron, Ohio, United States of America; 3 College of Public Health, Department of Biostatistics, Environmental Health Sciences and Epidemiology, Kent State University, Kent, Ohio, United States of America; 4 College of Podiatric Medicine, Department of Preclinical Sciences, Kent State University, Kent, Ohio, United States of America; Clemson University, UNITED STATES

## Abstract

The PubMed database offers an extensive set of publication data that can be useful, yet inherently complex to use without automated computational techniques. Data repositories such as the Genomic Data Commons (GDC) and the Gene Expression Omnibus (GEO) offer experimental data storage and retrieval as well as curated gene expression profiles. Genetic interaction databases, including Reactome and Ingenuity Pathway Analysis, offer pathway and experiment data analysis using data curated from these publications and data repositories. We have created a method to generate and analyze consensus networks, inferring potential gene interactions, using large numbers of Bayesian networks generated by data mining publications in the PubMed database. Through the concept of network resolution, these consensus networks can be tailored to represent possible genetic interactions. We designed a set of experiments to confirm that our method is stable across variation in both sample and topological input sizes. Using gene product interactions from the KEGG pathway database and data mining PubMed publication abstracts, we verify that regardless of the network resolution or the inferred consensus network, our method is capable of inferring meaningful gene interactions through consensus Bayesian network generation with multiple, randomized topological orderings. Our method can not only confirm the existence of currently accepted interactions, but has the potential to hypothesize new ones as well. We show our method confirms the existence of known gene interactions such as *JAK-STAT-PI3K-AKT-mTOR*, infers novel gene interactions such as *RAS- Bcl-2* and *RAS-AKT*, and found significant pathway-pathway interactions between the JAK-STAT signaling and Cardiac Muscle Contraction KEGG pathways.

## Introduction

With over tens of millions citations, PubMed, which offers free access to the National Library of Medicine’s MEDLINE database, contains a great wealth of biomedical literature records [1]. An investigator can use PubMed’s search capabilities to locate and read publications using simple data mining techniques such as keyword, author, year, and publication-name searches. The issue arises that with such a vast storage of information, to manually search and aggregate this information in order to discover potential relationships among the data requires an immense amount of time. In order to maximize the retrieval and use of the information available within PubMed, many post-processing computational tools have been developed. Plikus [2] provides a comprehensive group of third-party search interfaces [3–5] as well as systems that associate PubMed literature with ontology databases [6–8].

As PubMed provides a platform to access extensive amounts of information from biomedical literature, other data repositories such as GEO [9] and GDC [10] allow for the storage and retrieval of extensive amounts of genomic data from micro-array analysis [11, 12] and next-generation sequencing [13–15]. In part, the desire to determine genetic interactions using this data has led to the adaptation of commonly used bioinformatics methods including Neural Networks [16], Support Vector Machines [17], and Bayesian Networks [18, 19]. Additionally, past studies have inferred genetic interactions through the discovery of the co-occurrence of gene names within the abstracts [20, 21] and the linguistic analysis of relevant records acquired from PubMed [22]. We have created a system with which to combine the statistical power of Bayesian networks with the genetic information contained within citations accessible from the PubMed database in order to infer interactions among genes. Current genetic interaction software systems include Reactome [23, 24], Ingenuity Pathway Analysis (IPA) [25], the KEGG pathway database [26, 27], BioGrid [28], and IntAct [29]. Reactome is a curated, peer-reviewed database of genetic pathways composed of an organisms complete set of genetic reactions [30]. IPA has several features including pathway analysis, predictive causal analysis, and next-generation sequencing data analysis, all of which utilize their Ingenuity Knowledge Base; a repository of expert-curated biological interactions and functional annotations. While similar to these, our system does not rely on the input of expert biologists curating experiment data or publication information.

The structure of a Bayesian network can be representative of the interactions among genes within a biological pathway. Interactions among the genes are represented using directed edges among nodes in the network [31]. Constructing Bayesian networks can be a complex and computationally intensive process. The amount of data available within PubMed is large and the search space involved in Bayesian network creation can grow exponentially without imposing restrictions on the network.

Methods involved in network construction generally fall into two categories: constraint-based and score-based. Constraint-based construction attempts to reduce the search space by placing restrictions on the structure of the network. The restrictions imposed on the network depend on the conditional influence nodes have on one another. This reduction of the search space allows for faster computation of potential network structures, but the potential for compounding error increases with each reduction [32]. Score-based methods, selecting the network structure based on a maximum score, have a higher degree of precision. The issue with higher precision scoring methods are their higher computational complexity, limiting score-based construction to low-dimensional data.

In order to reduce the computational runtime of Bayesian network creation, Sriram suggests a combination of both constraint- and score-based construction utilizing the K2 algorithm [33], KEGG pathway information and an initial topological ordering of the genes that reduces the initial search space to a smaller, low-dimensional set of data for use with a scoring method. One possible caveat of using the K2 algorithm is the potential bias introduced from the initial topological input. The utilization of prior knowledge to construct these topological inputs as well as the generation of consensus networks from multiple Bayesian networks can help mitigate this bias [32]. Consensus networks have been utilized in the past to combine data from several networks into one; a standard use case being phylogenetic trees [34, 35]. Consensus networks have been utilized with Bayesian networks as well [36], but differ from ours in that they retain the directed edges and structure of a Bayesian network, whereas our method utilizes every edge and removes directed aspect.

We applied the idea of consensus network generation utilizing the K2 algorithm with data mined from the PubMed database. When using K2, a topological input is given, restricting the possible parents each node in the network can have. This topological ordering is normally constructed using expert knowledge of the data being examined. This can be accomplished by curating publications and experiment data. Additionally, the use of natural language processing like that integrated into Chilibot [22] can be a potential solution to generating knowledge of the data. Instead, in order to remove the need for expert curators and natural language processing, we expand the number of topological orderings supplied from several to many, and increase their potential orders to include not a specialized group of inputs, but an all-encompassing group of possibilities. This allows us to create extensive consensus networks comprised of a large number of individual Bayesian networks in order to remove this potential bias. In addition, the expanded input allows us to introduce the concept of network resolution. What we are calling network resolution is the ability to adjust the sensitivity of the findings from a broad, inclusive set of connections to a narrow, focused group with a higher potential to be true interactions. Using our method, investigators can not only confirm the existence of currently accepted genetic interactions through use of high-resolution networks, but can also hypothesize novel interactions by lowering the resolution to reveal additional, potential interactions.

## Materials and methods

### Datasets

**Fig 1:**
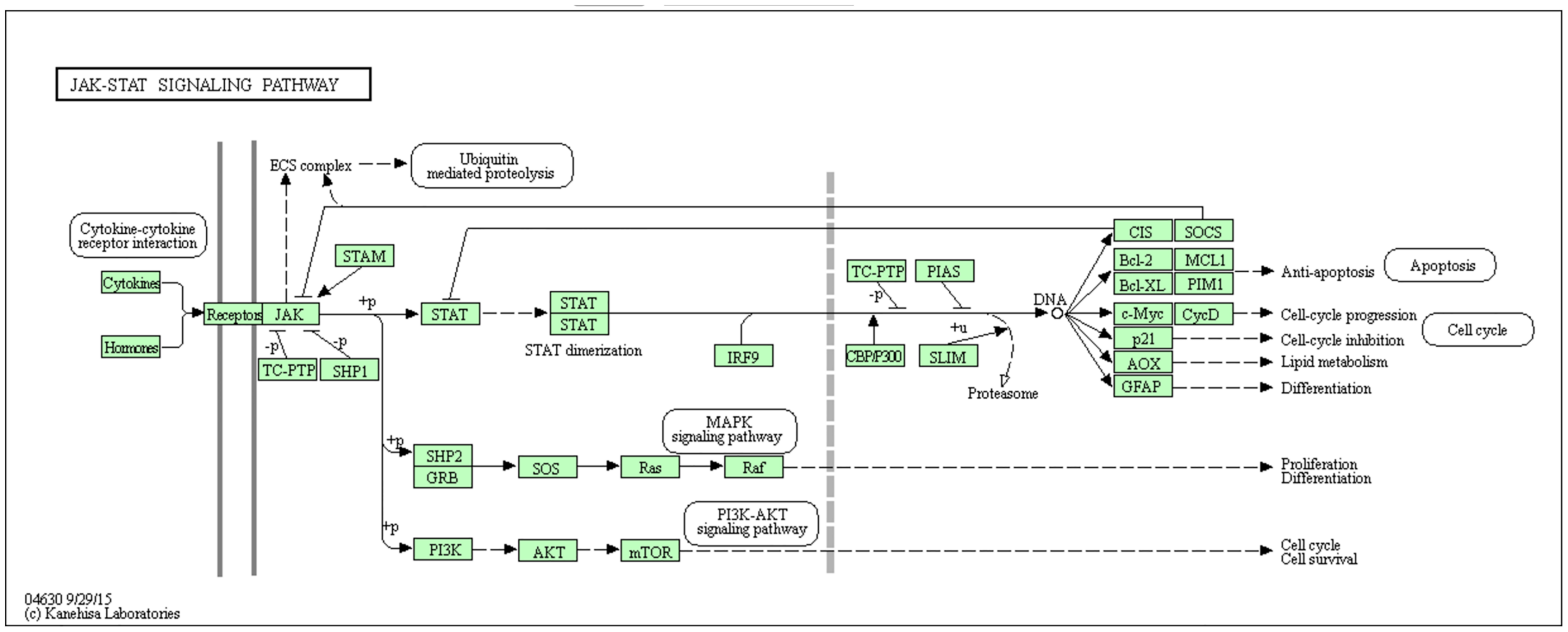
The JAK-STAT signaling KEGG pathway. The JAK-STAT signaling KEGG pathway shows the known interactions within the JAK-STAT signaling cascade.

**Fig 2:**
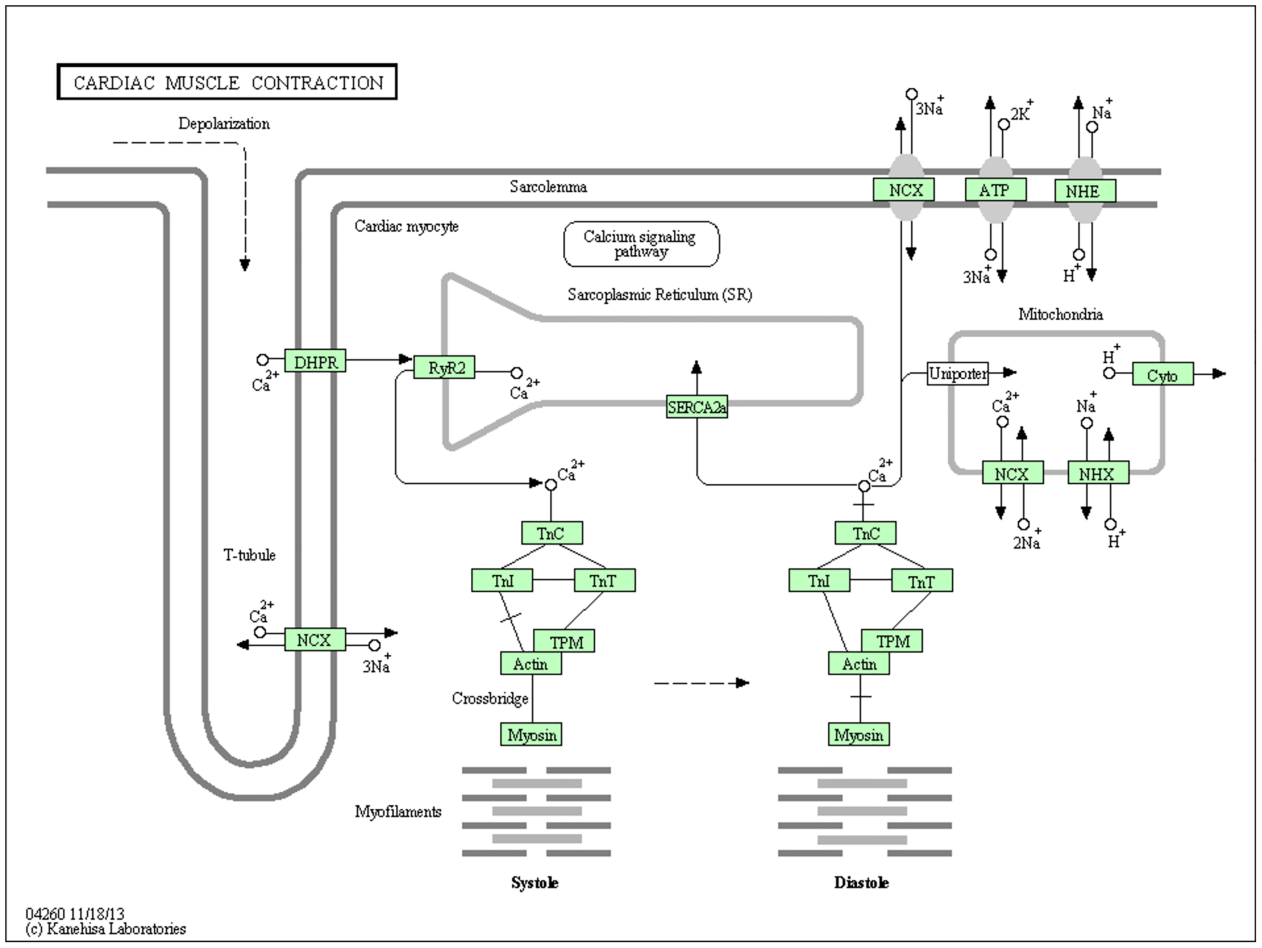
The Cardiac Muscle Contraction KEGG pathway. The Cardiac Muscle Contraction KEGG pathway shows how a ca2+ influx induces cardiac muscle contraction.

In order to create our Bayesian networks, we constructed a set of prior knowledge about the genes groups we were interested in. KEGG pathways present a set of molecules involved in a biological system and an overview of their interactions in a sequence of coordinated events. We created two separate datasets in order to examine both the stability of our method across multiple numbers of topologies and differing sample sizes as well as the functionality of the method and its output. In this study, we used the gene groups in the JAK-STAT signaling (Fig 1) and Cardiac Muscle Contraction (Fig 2) pathways as constraints to reduce the search space and explore the relationships among those presented in PubMed. The JAK-STAT signaling pathway contains 31 functional gene groups putatively involved with signaling for development and homeostasis in mammals. We focused on the key molecules involved in the signaling cascade. The molecules in the extracellular space such as hormones and cytokines, as well as receptors, were not included in any network construction. The Cardiac Muscle Contraction pathway contains 13 functional gene groups putatively involved with the contraction of the heart. Three negative control genes were also included: *SCF*, *NOS*, and *AC5*.

For both data sets, composing the prior knowledge about the selected gene groups involved data mining PubMed for publications that contained a member from at least two separate gene groups. The selected members for each gene group were treated as a single entity when searching, and a successful search for any of the members within a group was treated as a successful search for the entire group. Each publication that met this requirement became a row in the initial Bayesian network prior knowledge publication list. After list compilation, regular expressions were used to examine the abstracts within the list to create the prior knowledge matrix, searching again for the presence of members from any of the gene groups. This matrix indicated the presence or not of each of the gene groups within each publication. Parentheses, spaces, dashes, and brackets were allowed at the beginning and end of the search terms, and periods, commas, colons, and semi-colons were allowed at the end only.

The columns of the resulting matrix consisted of the gene groups, and the rows were successfully searched publications. The value of the intersection of publication and gene group within the matrix was determined by whether the publication contained mention of the gene group within its title or abstract. If a member of the group was present, the value of the matrix at that row-column intersection was set to ‘1’. A value of ‘0’ was given for intersections where the group was not present within the abstract. During Bayesian network generation this matrix was used to represent prior knowledge about the interaction among genes of interest; the presence of two or more genes in the same publication can indicate an interaction among them.

**Fig 3:**
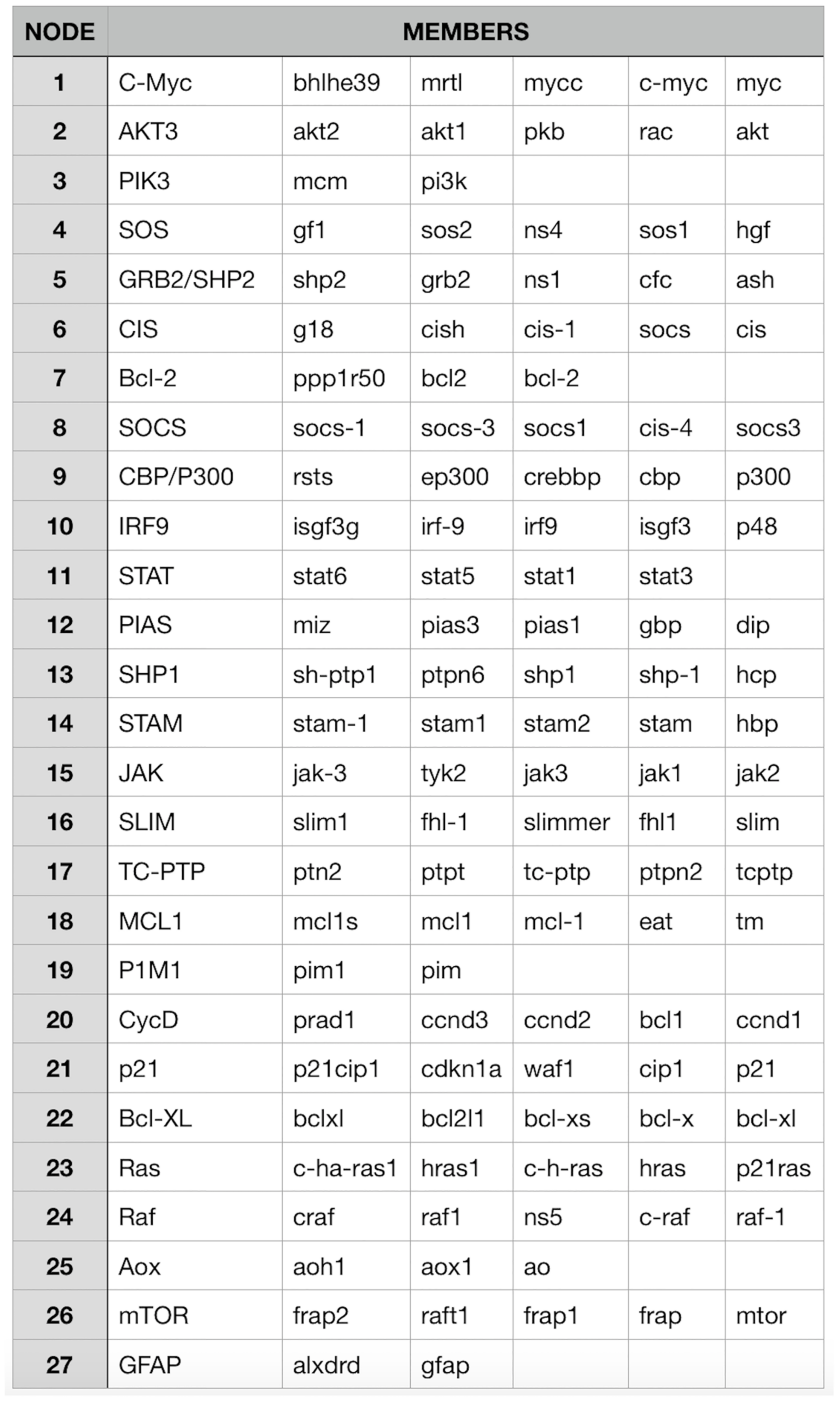
Consensus network creation. Using the functional gene groups from the JAK-STAT signaling pathway, PubMed searches were used to calculate the number of occurrences for each gene and its aliases within publication abstracts. The top five most commonly observed members from each functional gene group were selected for use as members of the data set. The node numbers match each gene group to a column number within its prior knowledge matrix (see S1 File).

The first data set (DS1), used to examine the stability of our method across different topologies and sample sizes, was created from functional gene groups within the JAK-STAT signaling pathway only. Because each group can contain multiple genes, and each gene can have multiple aliases, a list of all genes and their associated aliases for each gene group was used to create a list of members associated with the gene group. To reduce the number of members to those which are most commonly found within literature, members were sorted by the number of times they were found within the title or abstract of any piece of biomedical literature within PubMed. The top five most commonly observed members from each functional gene group were selected for use (see Fig 3). PubMed initial searching yielded a list of 42,600 publications for which the title and abstract were downloaded and analyzed. Matrix creation yielded a prior knowledge set with 27 columns and 42,600 rows (see S1 File). The node numbers from the member list match each gene group to its respective column number within the prior knowledge matrix.

**Fig 4:**
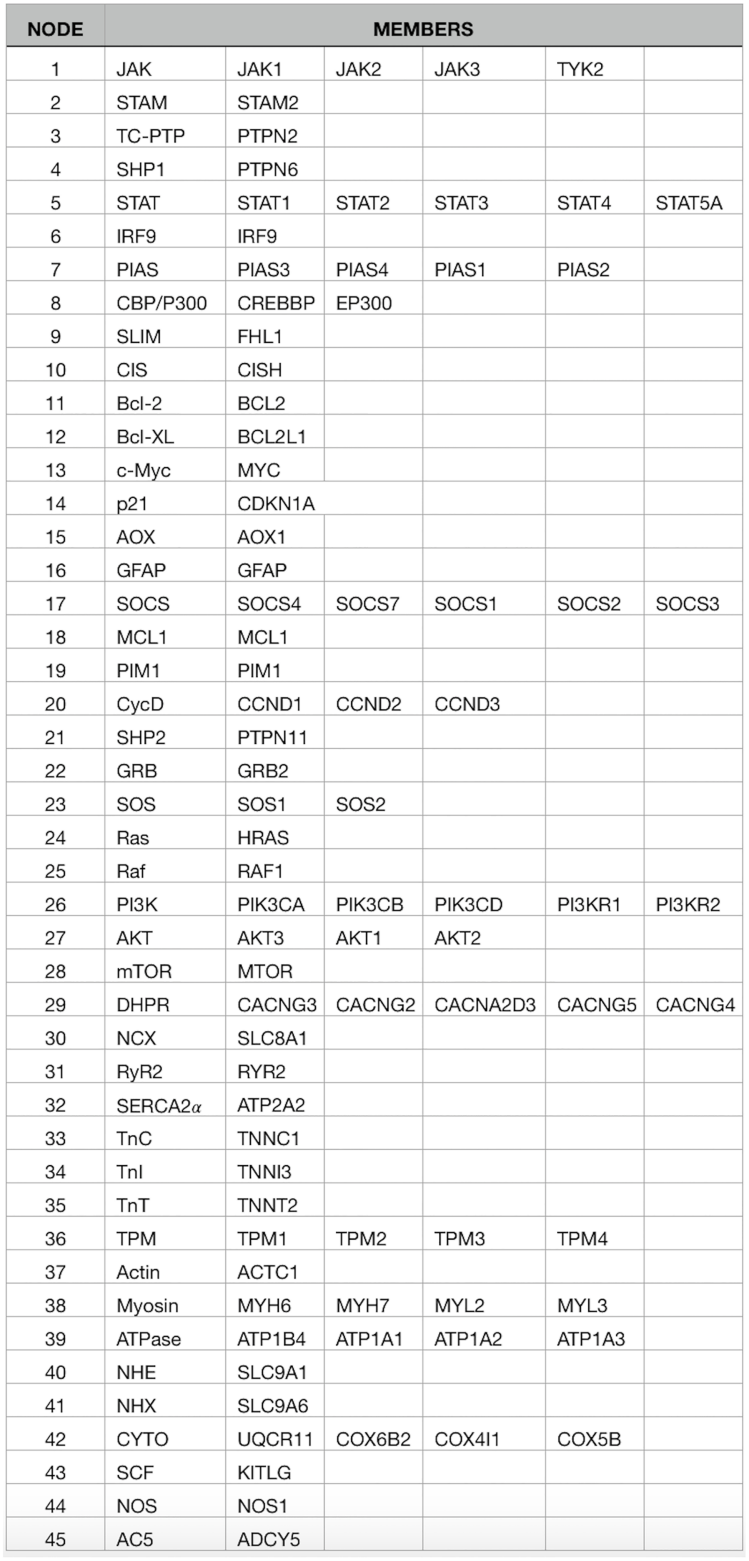
Consensus network creation. Using the functional gene groups from the JAK-STAT signaling and Cardiac Muscular Contraction pathways as well as three randomly selected genes, *SCF*, *NOS*, and *AC5*, 45 genes were selected as members of the functionality data set. The node numbers match each gene group to its respective column number within the prior knowledge matrix (see S2 File).

The second data set (DS2), used to examine the functionality and output of the method, was created using functional gene groups from the JAK-STAT signaling and Cardiac Muscle Contraction KEGG pathways as well as three randomly selected negative control genes: *SCF*, *NOS*, and *AC5*. Within this data set, each gene group’s members were selected using the gene group name displayed on the KEGG pathway as well as the first (up to) five genes indicated by the KEGG database. No additional aliases for genes were included (see Fig 4). PubMed initial searching yielded a list of 96,594 publications for which the title and abstract were downloaded and analyzed. Matrix creation yielded a prior knowledge set with 45 columns and 96,594 rows (see S2 File). As before, the node numbers from the member list match each gene group to its respective column number within the prior knowledge matrix.

### Generation of Bayesian networks

Bayesian networks are comprised of a directed acyclic graph (DAG) in which the nodes represent random variables from the domain and an edge between two nodes represents a dependency between those variables. As the number of nodes within the network increases, the search space for Bayesian network learning grows exponentially. The K2 algorithm was used to reduce this search space. Given a specific starting node, the topological input used by K2 was created using a random permutation of the remaining nodes in the network. To score potential edges within the network, the marginal likelihood of the graph containing an edge was computed given the publication data within the input matrix. A maximum of five parents per node was used to reduce the density of edges within the graph. The resulting graphs were represented with adjacency matrices containing directed edges between nodes (see S3 and S4 Files).

### Consensus network construction

**Fig 5:**
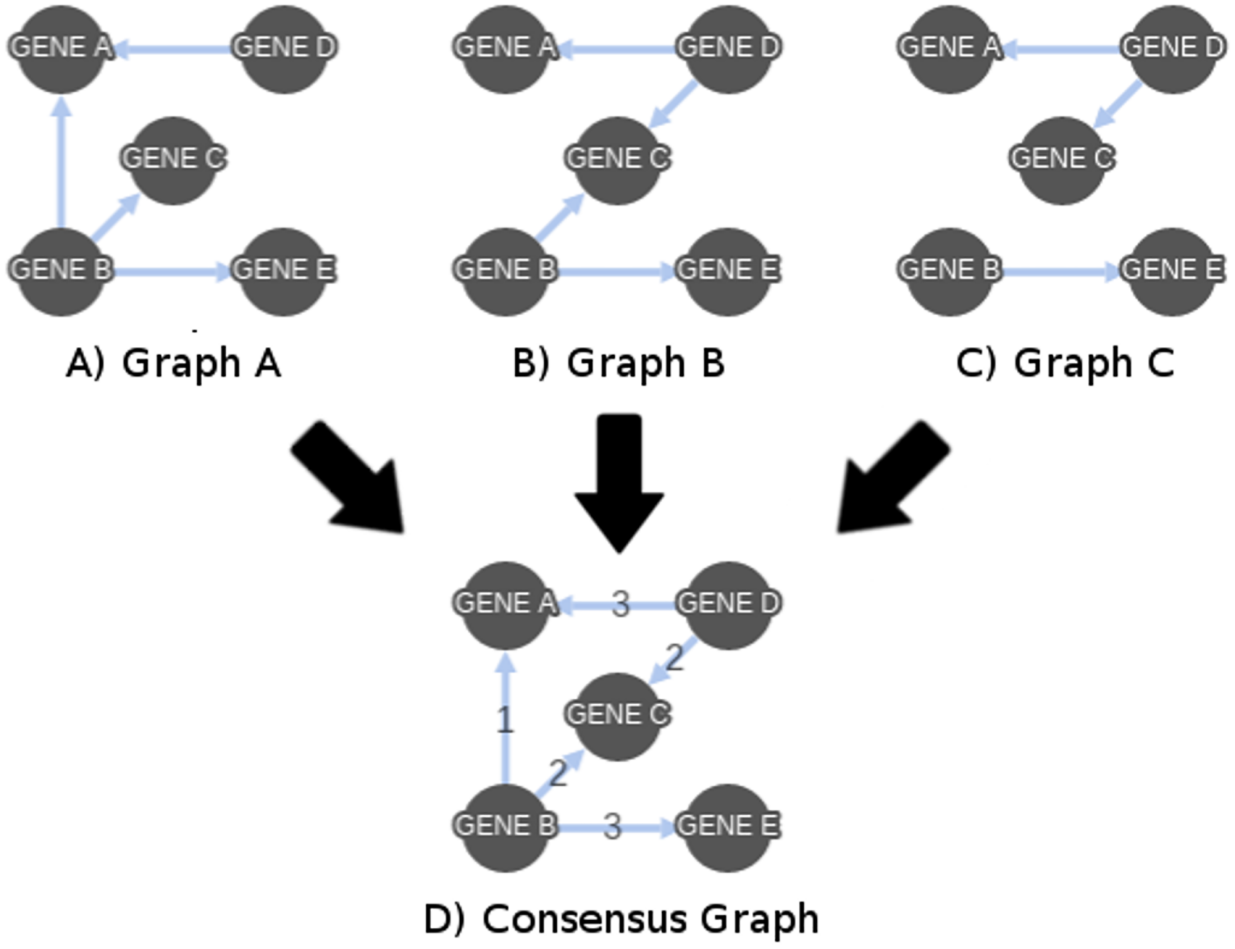
Consensus network creation. Graphs A, B, and C are used to create a consensus graph (D). The sum of the edge weights of each graph are used to determine the edge weight in the final graph.

**Fig 6:**
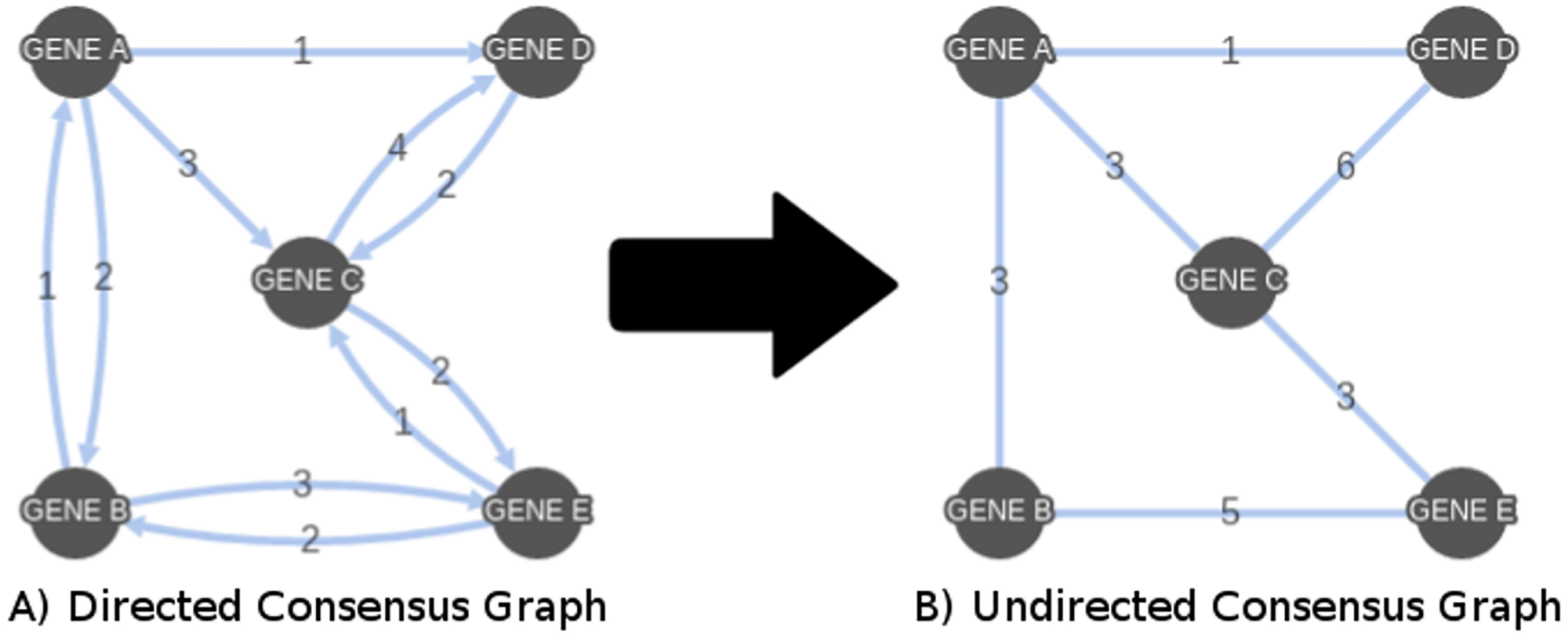
Removing bi-directionality from consensus networks. Bi-directionality is removed from directed consensus networks by adding the edge weights from both directions for each set of nodes in (A) and using an undirected edge with the new total in (B).

Since each node given within a topological order is limited to the prior nodes when looking for potential parent nodes, the topological input for K2 has the potential to introduce bias into the generated Bayesian network. In order to reduce this bias, multiple Bayesian networks were generated using randomized topologies that begin from every node within the network. From DS1, for each of the 27 functional gene groups, a number of randomized topologies, ranging from 4 to 100, were created. This resulted in the number of Bayesian networks created to be between 108 and 2700. The Bayesian networks generated using these topologies and their resulting adjacency matrices were combined, summing the total number of graphs containing each edge and creating a single network where edge weights represent the collection of these summations (see Fig 5). By storing the resulting network within an adjacency matrix, the combination of directed graphs has the potential to have directed edges in both directions between two nodes. This bi-directionality was removed by adding the matrix to the transposition of itself and removing edges in the lower left triangle. The result, a consensus network, is a single, undirected graph with the weight of each edge being the number of individual Bayesian networks containing it (see Fig 6). The consensus network for DS2 was created in the same manner as above, using eight topologies only, utilizing 360 Bayesian networks.

### Consensus network resolution

**Fig 7:**
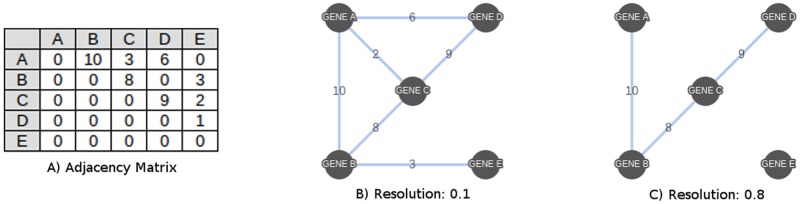
A consensus network at different resolutions. (A) The adjacency matrix for a consensus network created from 10 separate Bayesian networks. (B) The graph of the consensus network with a resolution of 0.1. (C) The graph of the consensus network with a resolution of 0.8. The resolution determines which edges to include by dividing the edge weight by the largest weight of any single edge in the network and removing those which do not exceed the cut-off.

By combining multiple Bayesian networks into a single consensus network, an edge with a larger weight (meaning it was present in a significant number of individual Bayesian networks) can be interpreted as having a higher potential for representing a true interaction between nodes. Because of this, we introduce the concept of edge resolution. The resolution of an edge, ranging from 0 to 1, is calculated by dividing its weight by the largest weight of any single edge in the network. An edge resolution threshold can then be introduced, and removing edges below differing thresholds will yield different interpretations. For example, high-resolution networks will contain edges that were present in only a small number of Bayesian networks, while low-resolution networks will contain edges present in a large number of networks (Fig 7). All testing of consensus networks was done on a range of ten resolutions from 0.1 to 1.0 in order to characterize results across all resolutions.

### Network stability across sample sizes

Depending on the search criteria used, sample sizes for publications retrieved from PubMed differ greatly in number. For consensus network generation via publication data to be considered stable across a broad range of sample sizes, we used Cohen’s kappa coefficient to determine if networks created with relatively smaller sample sizes would infer the same relationships as those created with a larger dataset. Cohen’s kappa coefficient is a statistic used to determine the chance-corrected proportion of agreement between judges classifying items into qualitative categories [37]. First, we randomly split DS1 into 10 sections of 4620. Second, for each test section, two consensus networks were generated: a test network created using the test section, and a predicted network was created using the remaining nine sections. Finally, for each pair of test and predicted networks, Cohen’s kappa coefficient was calculated for each of the ten testing resolutions (0.1–1.0). The kappa calculation was used to determine if each pair of networks agreed on the set of edges that composed their consensus networks.

### Stability across topological input sizes

The number of topological orders used to create a consensus network can be severely limited by the resources available with which to perform the calculations. The runtime of network creation grows linearly as the number of topologies increases. The question then arises, does a consensus network created with a smaller number of topologies differ, and by how much, than one created using more topologies? In order to test if consensus networks derived from differing numbers of topologies would yield the same consensus network, using DS1, a consensus network was built from differing numbers of random topologies, ranging from four to 100 by fours. For each successive pair of networks (four and eight, eight and 12, … 96 and 100) as well as the pair including four and 100, we calculated Cohen’s kappa coefficient to measure agreement between each of the 10 test resolutions (0.1–1.0).

### Functionality and inferred interactions

Using a prior knowledge data set that contains only the functional gene groups from a single KEGG pathway has the potential to make inferences within that pathway but lacks the ability to infer novel interactions with genes outside of the pathway. Having members from two different KEGG pathways as well as three negative controls, data set DS2 was used to verify the ability of our method to not only confirm existing intra-pathway interactions, but also infer pathway-pathway interactions, confirm a segregation of interactions between multiple pathways, and exhibit a lack of interactions among the three negative control genes. A consensus network was created using eight random topologies from each gene group, resulting in 360 Bayesian networks.

### Implementation and source code

The methods described in this experiment were performed using the following R packages: RISmed [38], KEGGREST [39], and KEGGgraph [40]. All Bayesian and consensus network generation for DS1 was done on the Blue Waters petascale machine at the University of Illinois at Urbana-Champaign utilizing 1024 Cray CPU-only XE6 nodes, consisting of two 16-core AMD processors with 64 GBs of RAM. [41]. Network generation for DS2 was completed on a 32-core Intel machine with 64 GBs of RAM. The source code of the software presented is freely available in the web repository GitHUB: https://github.com/Timer/bayesian-learning.

## Results

We analyzed resultant consensus networks generated by data mining publications from the PubMed database. The prior knowledge matrix for Consensus network creation for DS1 consisted of the following: 42,600 rows (representing the publications in which the abstract and/or title contained at least 2 of the members of the 27 functional gene groups in the JAK-STAT signaling KEGG pathway) and 27 columns (each representing the presence, or lack of, the functional gene group within the current row’s publication). Using DS1 we analyze the stability of our method across differing sample sizes and the stability of our method with a varying number of topological orders. Using DS2 we analyze the functionality and the resulting inferred interactions. Consensus network creation for DS2 consisted of the following: a prior knowledge matrix consisting of 96,594 rows (representing the publications in which the abstract and/or title contained at least 2 of the members of the 45 functional gene groups within the JAK-STAT signaling and Cardiac Muscle Contraction KEGG pathways as well as three negative control genes) and 45 columns (each representing the presence, or lack of, the functional gene group within the current row’s publication).

### Network stability across sample sizes

**Fig 8:**
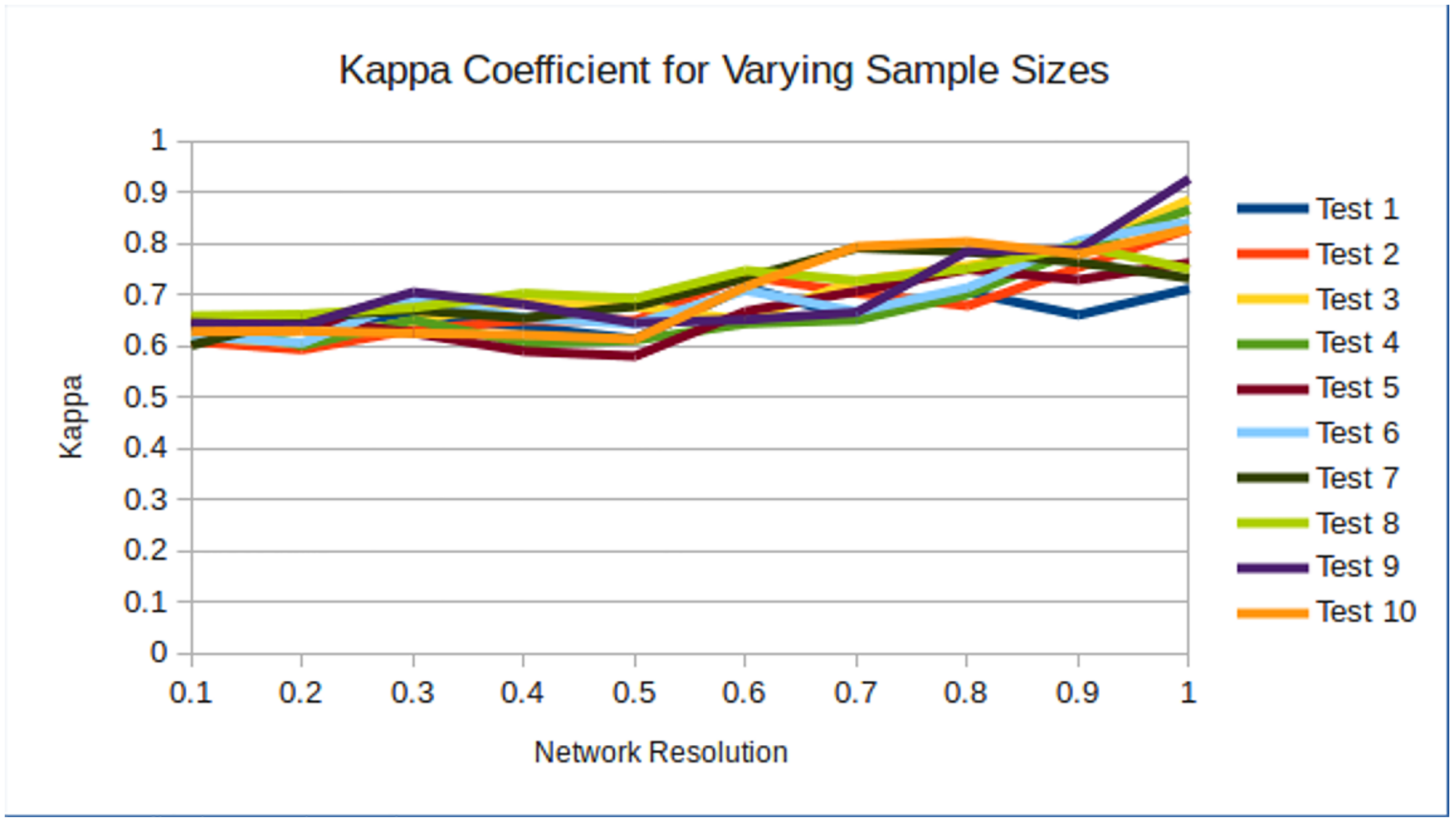
Kappa coefficients across network resolutions for differing sample sizes. When comparing consensus networks of differing sample sizes, for each of the 10 tests cases, Cohen’s kappa coefficient was calculated for 10 network resolutions ranging from 0.1 to 1.0.

Determining the stability of our method across different publication sample sizes was conducted by dividing the publication samples into 10 separate test sets and using them to create consensus networks, yielding 10 sets of edges representing inferred gene interactions. For each test case, the remaining publication samples were used to create a predicted consensus network, which would act as the network with which to test for agreement. For each of the 10 tests cases, Cohen’s kappa coefficient was calculated for 10 network resolutions ranging from 0.1 to 1.0. Fig 8 shows the kappa coefficients for tests 1 through 10, across the range of network resolutions. The full set of results showing the kappa coefficient for each resolution within each of the 10 tests can be found in S5 File.

### Stability across topological input sizes

**Fig 9:**
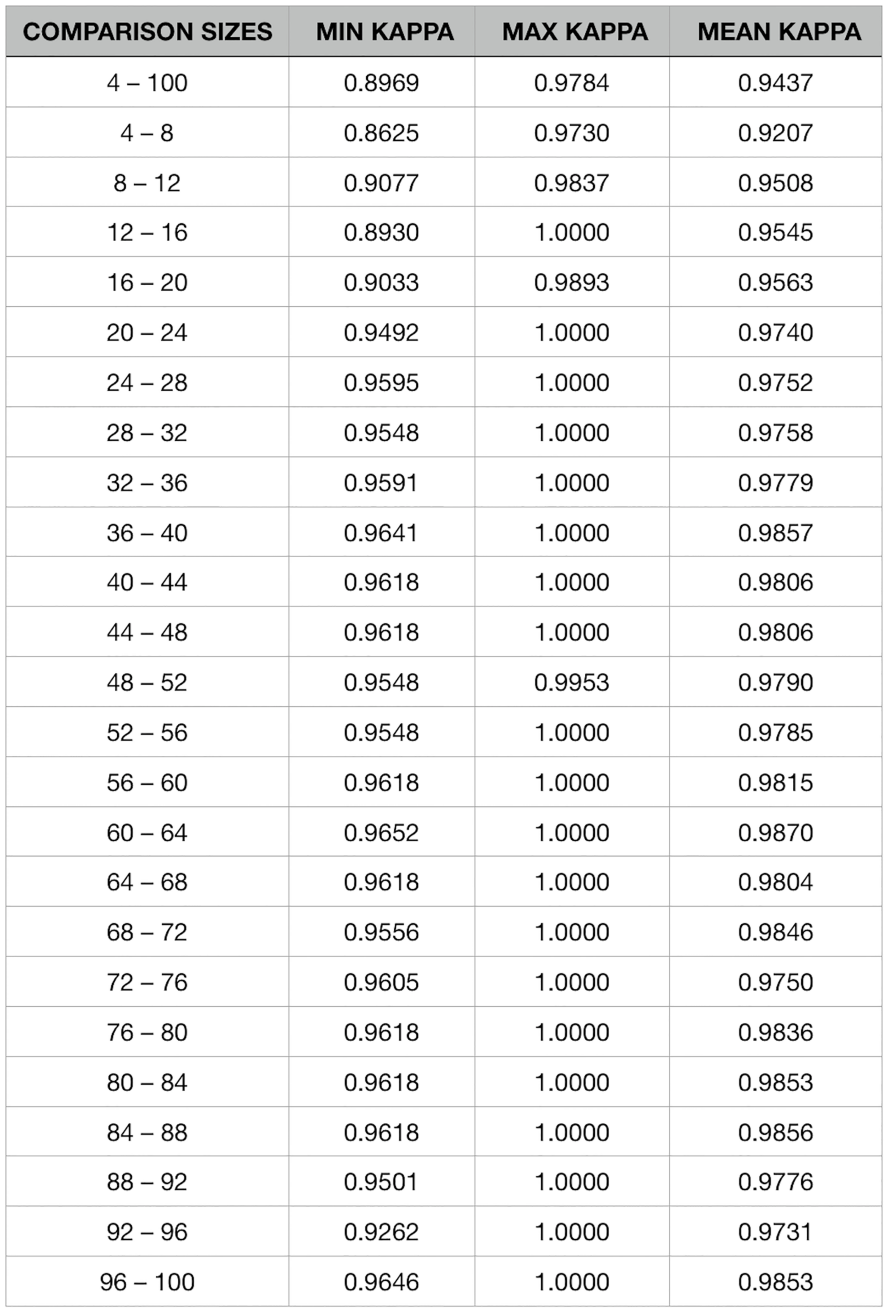
Kappa coefficients across network resolutions for differing sample sizes. The minimum, maximum, and mean kappa coefficients for each pair of networks created with varying sample sizes. It can be seen that variations in the sample size inputs for Bayesian network construction results in similar consensus networks.

With the high computational cost of generating Bayesian networks, and in turn consensus networks, it is expected that the number of topologies randomly generated for Bayesian network creation will vary depending on the resources available to the investigator. With this in mind, we tested the agreement between networks generated with different numbers of topological inputs. Topological input sizes ranging from four to 100 per node, increasing by four with each iteration, were created in order to compare their resulting consensus networks. Each network was compared with the network created by the next largest topological input size, and the networks created with the smallest and largest number of inputs were also compared. Cohen’s kappa coefficients were calculated for 10 network resolutions, from 0.1 to 1.0, and were used to determine the agreement between each set of networks, for each level of resolution. Fig 9 contains the comparison sizes as well as the smallest, largest, and mean kappa coefficients for each set of comparisons. The full set of results, showing the kappa coefficient for each resolution within each input size comparison can be found in S6 File.

## Discussion

With regard to network stability, if our method is stable across a wide range of sample sizes, the kappa coefficient should show agreement between each resolution for each test case. When testing the agreement between varying sample sizes, the kappa coefficients are uniform across all test and resolutions, with the lowest being 0.5802 at a network resolution of 0.5 (see S5 File). The nature of a lower network resolution allows for less consensus being needed to include an edge as an inferred interaction, therefore, the upward trend of the kappa coefficients mirrors the expectation of growth as network resolution increases. Using Cohen’s suggested interpretation of the kappa coefficient, all test cases show at least moderate agreement (0.41 to 0.60), all tests for resolutions above 0.5 show substantial agreement (0.61 to 0.80) or higher, and all tests at resolution of 1.0 are considered to have almost perfect agreement (0.81 to 0.99). These results indicate that at lower resolutions a smaller sample size will have at least a moderate agreement on which interactions are inferred, and as the resolution increases, so will the agreement between small and large sample sizes.

With the high computational cost of generating Bayesian networks, and in turn consensus networks, it is expected that the number of topologies randomly generated for Bayesian network creation will vary depending on the resources available to the investigator. With this in mind, we tested the agreement between networks generated with different numbers of topological inputs. In all cases, including the one between the smallest and largest number, the kappa coefficient is in the ‘almost perfect’ agreement range (0.81 to 0.99) and the max kappa among resolutions in five of the tests was considered perfect (1.0) (see S6 File). This indicates that the inferred interactions will be extremely similar regardless of the number of topological inputs. A slight upward trend can be observed in the mean kappa coefficient as the number of topological inputs increases, indicating there is a slight increase in stability as more Bayesian networks are utilized.

A driving focus of empirical research is to derive a causal relationship between a stimulus and a downstream molecule or receptor that can then drive a behavioral response, physiological change, and or produce an epigenetic alteration [42]. Paramount to driving the next generation in bioinformatics is the use of computational models to confirm current molecular pathways but also to help derive putative functional relationships previously unknown [43]. The data gathered within this study has focused on leveraging consensus networks to derive causal relationships that aim to uncover pathway interactions beyond KEGG curated pathways. Specifically, we tested our method on the current state of biochemical signaling knowledge on JAK-STAT signaling with an aim to distill them to core relationships without the use of significant computational resources. As shown in Fig 9, the optimal strategy for the number of topological inputs is essentially four (which is far less than 100+) and results in a significant reduction in computational power and in turn a reduction in computational time.

**Fig 10:**
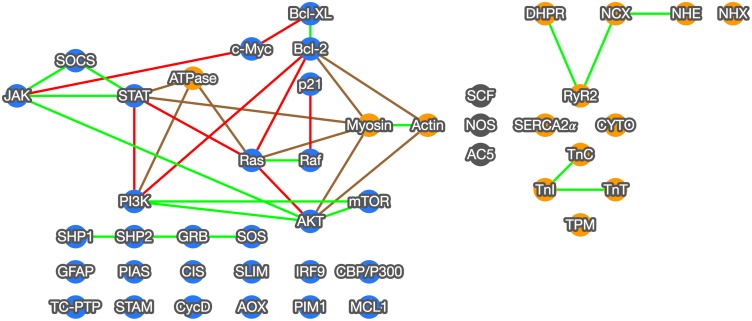
Consensus network output. Mining genetic information from abstracts cited within PubMed, our method generated a consensus network using large numbers of Bayesian networks representing potential genetic interactions within the JAK-STAT signaling (blue nodes) and Cardiac Muscle Contraction (orange nodes) KEGG pathways. Gray nodes are randomly selected negative controls. This graph shows the consensus network at a resolution of 0.9. Edges between genes indicate an inferred relationship. Green lines indicate a conserved interaction between the consensus network and the KEGG pathway, red lines indicate a novel interaction within the pathway, and brown lines indicate pathway-pathway interactions.

As shown in Fig 10, using a resolution of 0.90, 35 inferred interactions are present within the consensus network. The nodes are displayed to mirror their biochemical pathway positioning within the KEGG database for JAK-STAT signaling and Cardiac Muscle Contraction pathways. Illustrated in blue, the JAK-STAT signaling KEGG pathway is signaled through cytokines. Once bound to its receptor, Janus Kinase (*JAK*) based signaling is initiated resulting in subsequent downstream signaling. Illustrated in orange, The Cardiac Muscle Contraction and three randomly chosen negative control genes, shown in gray, are included to illustrate correct functioning of the algorithm and if pathway-pathway interactions are present. Shown in green, we found 18 similar interactions between the two KEGG pathways and our consensus network, with eight putative novel direct interactions shown in red. Nine pathway-pathway interactions were discovered as shown in brown.

The mammalian JAK-STAT signaling pathway has been extensively studied for the past twenty years [44] in mammals through gene knockout models, crystallography studies, and the use of antibodies for detection of specific isoforms and pharmokinetic studies to unravel disease pathologies [45]. JAK-STAT signaling is intimately associated with cell membrane cytokine receptors and at its core signaling, there are over 35 different cytokine receptor combinations with over 37 cytokines they respond to [46]. Specifically in mammals, there are four *JAKs* and seven *STATs* that are used in combination by over 50 cytokines [47]. These redundant, yet varying combinations allow for tissue specific responses. Essentially most all cytokine receptors signal through *JAKs* and their subsequent downstream effector molecules, signal transducer of activation (*STAT*) proteins, while others signal through *MAP* Kinase cascade. Nonetheless, we focused this study within the proximal JAK-STAT signaling pathway.

As JAK-STAT pathways are involved in a plethora of Metazoan biochemical processes, most are focused in cellular growth, differentiation, survival, and resistance to cell death [48]. Interestingly, we did include ubiquitin mediated proteolysis which is involved in tagging cells for degradation, as well as *MAPK*, which can manipulate cellular proliferation and growth through alteration of cellular transcription [49]. Additionally, the *PI3K-AKT* signaling pathway is also involved in cytokine signaling, involved in modifying cell survival, proliferation, and even glucose metabolism [50]. Thus the JAK-STAT pathway has a multi-pronged approach to modifying cellular behavior and our consensus network identified three distinct interactions, and their corresponding nodes were identified as conserved between the KEGG pathway and our consensus network; *JAK-STAT-PI3K-AKT-mTOR*, *JAK-STAT-SOCS*, and *SHP1-SHP2-GRB-SOS* (Fig 10).

### Conserved pathway interactions

One of the largest conserved interactions between our consensus network and KEGG was *JAK-STAT-PI3K-AKT-mTOR* Fig 10. This pathway is responsible for a significant number of interactions which will be described below. With *JAK* responsible for phosphorylating STAT, and *STAT* dimerization translocated to the nucleus to cause transcriptional changes, JAK and *STAT* are intimately involved. *STAT* also has an interaction with PI3K signaling [51]. PI3K is a lipid kinase that controls signaling and cell regulation [52]. For example, in human tumor cell lines, a proteomic study found a significant link between *PI3K* and *STAT3* in human cancer causing enhanced phosphorylation, with its effects reversible with PI3K inhibitors [53]. Despite being responsible for cell regulation, *PI3K-AKT* is also related to tumorigenesis [54]. With its concordant discovery in the early 1980’s, the *PI3K-AKT* pathway is a result of *PI3K* enzyme activation phosphorylating membrane inositol lipids, impacting cellular signal transduction [55, 56]. Once activated, this drives *AKT- PKB* kinases to change their conformation and phosphorylation. Activated *AKT* then moves to the nucleus, activating target sequences involved in cell proliferation, survival, growth, and angiogenesis [55]. One hormone that can specifically modulate this pathway is insulin, which modifies glucose uptake, among others [57], which is critical to cell growth. As tumor detection and mortality rates have slowly changed over the past decade, an enhanced vigor in therapeutic targets have revolved around the *PI3K-AKT* pathway for cancer drug discovery [58–60], specifically with the addition of genomic studies [61]. Ultimately, *mTOR* (mechanistic target of rapamycin) is the end kinase complex in our conserved interaction that is responsible for protein synthesis, autophagy, and cell survival which interestingly can be modulated by nutrients and growth factors as previously described above [62]. Collectively this cascade is critical to cell function and an inability to keep cellular proliferation and signaling in check can lead to disease and is why these genes act together.

Our consensus network also found a conserved signaling cascade between *JAK-STAT* and *SOCS*. Much of the JAK-STAT signaling pathway is related to internal cellular signaling, therefore, having signaling molecules in place to provide negative feedback is critical to halt runaway responses. The suppressor of cytokine signaling (SOCS) proteins include approximately eight intracellular proteins (*SOCS1-7*, and *CIS*) and function as E3 ubiquitin ligases that mediate the degradation of proteins [63, 64]. *SOCS* proteins were initially coined as repressors of cytokine signaling and loss of function or disruption in signaling lead to chronic inflammation and unchecked cellular growth [65, 66]. Thus, it is of no surprise that *JAK-STAT* and *SOCS* developed a conserved interaction in our consensus network as fidelity in signaling is critical to regulated cytokine signaling.

A third conserved interaction is *SHP1-SHP2-GRB-SOS*. *SHP1* and *SHP2* play critical roles in cellular growth, differentiation and cellular chemotaxis, but have also been implicated in cancer, neurodegeneration, and metabolic disorders such as diabetes [67]. *SHP1* and *SHP2* are cytoplasmic protein tyrosine phosphatases that work in concert but also oppose cellular cascades, with *SHP1* a negative transducer and *SHP2* a positive signal transducer [68]. Of note, a reduction in *SHP1* is observed in lymphomas and luekemias [69], whereas a reduction in *SHP2* or knockout of *SHP2* gene causes death mid gestation in mice [70], overall reduced *STAT* activation [71], and even leads to Noonan syndrome [72]. *SHP2* activity is a well-known binder of GRB2 [73], and *GRB2* binds to Son of Sevenless (*SOS*). This overall binding to a guanine nucleotide exchange factor is the mode by which reactivation of *RAS* is accomplished. This allows re-engagement of *RAS*, an oncogene, and *RAF* [74, 75]. Mutations in either *RAS* or *RAF* have been linked to several human cancers [76]. *SOS* helps to bind GTPases which are essentially molecular switches for cellular activity that hydrolyzes *GTP* to *GDP* plus phosphate that induces *RAS* activation and ultimately cellular proliferation and differentiation. Mutations in *SOS* can lead to hereditary gingival fibromatosis type 1 [53]. It is not surprising that some of the most talked about and well-known pathways have a very direct relationship and easily measurable tangibles as shown in these three examples. However, we also observed other interactions in our consensus network that can not be superimposed over the KEGG database pathway, which represent significant correlations within JAK-STAT.

### Novel pathway interactions

One of the most significantly novel JAK-STAT network interactions was *RAS-Bcl-2*. These two cell signaling genes are involved in cell fate with the former modulating *Bcl-2* activity and *Bcl-2*, involved in anti-apoptosis. Despite these two not being in a linear relationship within the JAK-STAT signaling cascade, previous functional relationships have been observed between *Bcl-2* and members of the Ras superfamily [77–79]. For example, though *Bcl-2* can be a ruler of anti-apoptosis and thus cancer cell survival, cross-talk interactions between small GTPases which are pharmacologically inhibited actually reverted death phenotypes of *Bcl-2* expressing cells, essentially modifying anti-apoptosis and thus providing an additional target for drug resistant cancers [78]. These poorly understood crosstalk interactions have only recently been identified [80] and our use of consensus networks has reaffirmed and also highlighted significant correlative protein interactions. Upstream of *Bcl-2* and *RAS* is a negative repressor of JAK-STAT signaling, (*SOCS*), which works to halt runaway activation of *JAK*.

Another unique novel interaction found within our consensus network was *RAS-AKT*. *RAS*, ras viral oncogene homolog, is a small GTPase that when bound with *GTP* is activated and used to transduce a signal primarily through *MAPK* signaling pathways (for example *RAF*) as shown in Fig 10. *AKT* (v-akt murine thymoma viral oncogene homolog) is a known oncogene and can cause rare genetic diseases such as Proteus Syndrome [81] and Cowden Syndrome 6 [82]. Other studies have found in *AKT*-null mice an increased sensitization to other cytokines like tumor necrosis factor and even genotoxic effects induced by gamma or ultraviolet radiation [83]. As both *AKT* and *RAS* lie within cell cycles responsible for cell survival and proliferation, respectively, it is not surprising that they may have an interaction. Wang and colleagues [84] found a putative functional relationship between *AKT-mTOR* and *RAS-MAPK* pathways in liver cancer. They identified a concomitant increase in *AKT* and *RAS* resulted in enhanced liver tissue carcinogenesis [85] whereas suppression of gene expression reduced cell growth. Thus these two pathways play an intimate co-functional role in cell survival and proliferation.

Lastly, another interaction identified within our Consensus network as novel within the confines of the KEGG pathway is *c-Myc* and *AKT*. *C-Myc* is an oncogene that is responsible for modulating transcription which impacts cell-cycle proliferation but is also critical to programmed cell death [86]. It is modulated by mitogenic signals and repressed by growth inhibitory signals [87]. Disruption in *c-Myc* results in significant reduction of cell growth [88] and has been linked to several cancers despite a half-life of 20–30 minutes [89]. A few studies have identified an inverse relationship between *AKT* and *c-Myc* [90, 91], with some finding that inhibition of *c-Myc* with MadMyc suppressed heptaocarcinoma development with a corresponding *AKT* induction [92]. These studies collectively identify that the significant interplay between unchecked cell proliferation and survival is what can ultimately lead to carcinomas. Just as additional research is needed to better unravel the shift from asymptomatic cancerous tissue to nutrient deprived and stressed cellular machinery that regulate cell apoptosis, cell proliferation, and other collective processes, these consensus networks point to conserved pathways and also uncover putative novel protein-protein interactions that can be further validated via genome-wide asociation studies, RNA-seq, and microarray analyses.

### Pathway-pathway interactions

In addition to the novel, community interactions inferred using our method, we also added three randomly chosen genes (Fig 10, highlighted in gray) as well as the Cardiac Muscle Contraction KEGG pathway, to test whether our algorithm could recapitulate conserved community interactions that are defined within KEGG and also resist the addition of randomly inserted genes and pathways. Based on data shown within Fig 10, our consensus network found several conserved, as well as unique, interactions within the KEGG pathways. With the addition of the Cardiac Muscle Contraction pathway, three gene groups found a significant pathway-pathway interaction. *ATPase* is an ancestral ATPase Na^+^/K^+^ pump that maintains an electrochemical gradient within muscle cells [93]. Much of this interaction is widely debated but more specifically it binds to transcription regulators like *SNW1*; a transcription regulator *SKIP* (Ski- interacting protein); *SMAD* family members like *SMAD7*, which is a known antagonist of *TGF-β* (Transforming growth factor-β); and even *PDPK1* kinase activity, which is responsible for activation of *AKT*, which is downstream of *PI3K*. Disruptions in Na^+^/K^+^ -ATPase function can lead to thyrotoxic periodic paralysis [94] and McArdle disease [95]. *Atpase* has a novel interaction with *PI3K* [96] and interestingly ouabain-induced signaling can impact the *EGFR-Src-RAS-ERK* pathway and also *PI3K1A-PDK-AKT* pathway [92, 97], with the latter resulting in hypertrophy solely in differentiated cardiac myocytes [98].

In addition to a Na^+^/K^+^ -*ATPase*, *Myosin* and *Actin* were also found to be present within the JAK-STAT portion of the consensus network. *Actin* is the most abundant protein present within the human body and is also one of the most conserved across a plethora of Metazoans. *Actin* and its isoforms are responsible for several functions, however it is most well-known for its cytoskeleton nature; within cardiac muscle contraction, thin filament *actin* is the scaffold upon which *myosin* contractions work [99]. As such, it is no surprise *actin* and *myosin* produce an interaction, however there are also interactions with *AKT* and *Bcl-2*. *Bcl-2*, as indicated previously, is a protein responsible for cellular metabolism, cell fate, mitochondrial function and ultimately cell proliferation with disruptions in its signaling causing B-cell follicular lymphoma [100]. Bcl-2 is also localized to the endoplasmic reticulum and regulates the control of Ca^2+^ levels which also influences *actin* cytoskeleton development and provides a cue in apoptosis [101]. Interestingly, *Bcl-2* is also found to enhance *F-actin* and *myosin* polymerization which results in inhibition of cell-cell adhesion and motility [102, 103]. Thus *Bcl-2’s* link to *myosin* and *actin* underline the importance of metastasizing cancers and reiterate the interrelatedness of the internal milieu within the human body. Collectively, these new pathway-pathway interactions reiterate the functional interrelatedness of biochemical pathways that are missed when just looking at a specific pathway alone and though our algorithm does not provide a causal relationship of interaction, the interaction is a powerful tool to identify significant community relationships among proteins.

## Conclusion

Our method of inferring gene interactions can be helpful to investigators in multiple ways. With our method, the confirmation of currently accepted gene interactions as well as the hypotheses of new interactions is possible. By using consensus networks created with multiple, randomly generated topological inputs, inferences can be made about gene interactions without prior knowledge of the gene functions themselves. Any group of genes can be examined for possible interactions, regardless of biological system, and even across organisms. The method of consensus network generation with randomized topological inputs could also be adapted for use with other sources of data: microarray, RNA-Seq, and mutation data. The more prior knowledge that is applied to the initial gene selection, the more precise and informative the results will be. Additionally, the current version of our text searching and parsing is very rudimentary; the addition of natural language processing could greatly increase the effectiveness of the entire process, even allowing the detection of specific interaction types. In its current form, our method uses R and Matlab to query and pre-process data, with a large parallelized, petascale system to generate Bayesian networks. We are moving towards releasing a web-driven front end that would allow investigators to use this system with their own data, integrated KEGG pathway data, and data from large experiment databases such as the GDC.

## Supporting information

S1 File Prior knowledge matrix for data set 1. A matrix representing the prior knowledge input used with creating our Bayesian networks. The matrix has 28 columns and 42600 rows. The rows consist of publications which contain at least two of the functional gene groups we are interested in. The first column is the PubMed ID of the publication represented by the current row. The remaining 27 columns represent the functional gene groups given in Fig 3. The value of these 27 columns are ‘0’ if the publication did not contain the column’s genes group, and ‘1’ if the group was present within the publications abstract.
(CSV)

S2 File Prior knowledge matrix for data set 2. A matrix representing the prior knowledge input used with creating our Bayesian networks. The matrix has 46 columns and 96,594 rows. The rows consist of publications which contain at least two of the functional gene groups we are interested in. The first column is the PubMed ID of the publication represented by the current row. The remaining 45 columns represent the functional gene groups given in Fig 4. The value of these 45 columns are ‘0’ if the publication did not contain the column’s genes group, and ‘1’ if the group was present within the publications abstract.
(CSV)

S3 File Consensus network adjacency matrix for data set 1. The adjacency matrix representing the consensus network generated with 42,600 publications, 27 functional gene groups, and 100 random topologies per group resulting in 2700 total Bayesian network inputs. Each row and column index represents the matching group from Fig 3. The numbers within the matrix represent the number of Bayesian networks that contained the edge connecting the group with the column group.
(CSV)

S4 File Consensus network adjacency matrix for data set 2. The adjacency matrix representing the consensus network generated with 96,594 publications, 45 functional gene groups, and eight random topologies per group resulting in 360 total Bayesian network inputs. Each row and column index represents the matching group from Fig 4. The numbers within the matrix represent the number of Bayesian networks that contained the edge connecting the group with the column group.
(CSV)

S5 File Sample size kappa coefficients. The Cohen’s kappa coefficient results for each pair of consensus networks used to test stability across sample sizes. There are 10 different tests in the file. Each test contains 10 network resolutions, each having its own set of four values used to calculate the kappa coefficient.
(XLS)

S6 File Topological kappa coefficients. The Cohen’s kappa coefficient results for each pair of consensus networks used to test stability across topological input sizes. There are 25 different tests in the file. Each test contains 10 network resolutions, each having its own set of four values used to calculate the kappa coefficient.
(XLS)
